# Humanized anti-CD25 monoclonal antibody treatment of steroid-refractory acute graft-versus-host disease: a Chinese single-center experience in a group of 64 patients

**DOI:** 10.1038/bcj.2015.33

**Published:** 2015-04-17

**Authors:** T Tao, X Ma, J Yang, J-Y Zou, S-M Ji, Y-S Tan, W Gong, F Du, J Xu, C-M Ye, X-W Tang, D-P Wu, S-L Xue

**Affiliations:** 1 Department of Hematology, Loufeng Branch of the First Affiliated Hospital of Soochow University, Suzhou, China; 2 Department of Hematology, The First Affiliated Hospital of Soochow University, Suzhou, China; 3 Hematopoietic Transplant Institute, Soochow University, Suzhou, China; 4 Jiangsu Institute of Hematology, The First Affiliated Hospital of Soochow University, Suzhou, China; 5 Collaborative Innovation Center of Hematology, Soochow University, Suzhou, China; 6 Department of Clinical Nutrition, The First Affiliated Hospital of Soochow University, Suzhou, China; 7 Suzhou Health College, Suzhou, China

Allogeneic hematopoietic stem cell transplantation (allo-HSCT) is a potentially curative therapeutic approach for hematologic malignancies. However, acute graft-versus-host disease (aGVHD), especially the refractory type, remains a serious barrier to successful allo-HSCT. Various second-line treatment regimens have been proposed; yet, to date, no standard treatment protocol has proven to be the best choice.^1^ The anti-CD25 monoclonal antibody (mAb) is one of the most used second-line treatment choices for steroid-refractory aGVHD.^2^ Unfortunately, China has limited experience with regard to the application of anti-CD25 mAb in the treatment of steroid-refractory aGVHD. Herein, we report our experience with humanized anti-CD25 mAb treatment in a group of Chinese allo-HSCT patients affected by steroid-refractory aGVHD.

Between December 2011 and April 2014, 352 patients were diagnosed with aGVHD at the Department of Hematology in the First Affiliated Hospital of Soochow University, and 64 patients did not respond to first-line treatment^3^ and they were enrolled into this study consecutively. The median time of steroid-refractory aGVHD onset after HSCT was 1.67 months (range, 0.73–14.2 months) in this group of patients. The institutional review board of the First Affiliated Hospital of Soochow University approved this study protocol and the signed informed consent was provided by the enrolled patients or their guardians if the patients were younger than 18 years old. The patient characteristics are summarized in Table 1, and the severity of aGVHD was graded according to the Keystone 1994 consensus criteria.^4^

On using standard-dose methylprednisolone (2 mg/kg/day) treatment, if aGVHD still progressed within 3 days or if it was not alleviated after treatment for 5–7 days, then the aGVHD is considered steroid refractory and second-line therapy can be given,^1^ and simultaneously, the steroid should be tapered.^5^ Humanized anti-CD25 mAb (Xenopax, CP Guojian Pharm, Shanghai, China) that has the same amino-acid sequence as daclizumab (Zenapax, Roche, Nutley, NJ, USA) was administered at a dosage of 1 mg/kg intravenously on days 1, 4, 8, 15 and 22.^6^ If the skin or gastrointestinal tract aGVHD did not respond to the systemic glucocorticoid treatment, topically worked glucocorticoid in ointment or capsule was given to avoid the exacerbation of aGVHD due to the tapering of systemic steroid, respectively.^7^

In our study, the responses of patients were concluded at day 28 after therapy initiation.^8^ Complete response (CR) was defined as resolution of all signs and symptoms of aGVHD in all organs without intervening salvage therapy; and partial response (PR) was an improvement of at least one stage in one or more organs without progression in any other organ. No response (NR) was defined as the absence of improvement or aGVHD progression within 28 days after therapy initiation.

Previous studies have demonstrated that daclizumab treatment for steroid-resistant aGVHD could produce a response rate ranging between 29 and 68%.^2^ However, our results were even more encouraging, a total response rate of 83% (53/64) was achieved, which included a CR rate of 58% (37/64) and a PR rate of 25% (16/64). Although a total response rate of 89% (31/35) achieved in steroid-refractory aGVHD patient subgroup with single organ involved is higher than that of 76% (22/29) achieved in multiorgan-involved patient subgroup, there is no significant difference between these two groups (*P*=0.15). Analyzed from the response data of the whole 64 patients population to anti-CD25 mAb therapy, gastrointestinal tract steroid-refractory aGVHD showed a significantly better response than skin and liver types, with a *P*-value=0.007 and <0.001, respectively, and skin steroid-refractory aGVHD showed a better response than liver type with a *P*-value <0.001. Supplementary Tables 1 and 2 show the response profile of the steroid-refractory aGVHD patients to anti-CD25 mAb treatment.

During the anti-CD25 mAb treatment phase, among all the 64 patients only 1 patient showed mild thrombocytopenia; another patient developed chills during the Xenapax infusion, after the infusion rate was slowed down and the administration of diphenhydramine, the symptom resolved quickly. No adverse effects on the liver or kidney and no other drug reactions were observed.

Chronic GVHD (cGVHD) is currently the leading cause of long-term morbidity and mortality following allo-HSCT.^9^ However, few data are available regarding the cGVHD epidemiology following refractory aGVHD patients receiving second-line treatment. In our patient group, 22 patients (22/64, 34%) developed cGVHD in total with a median time of 6.5 months (range, 3.6–14.0 months), in which 20 patients (20/22, 91%) were diagnosed with classic cGVHD, 2 patients (2/22, 9%) with overlap syndrome and 14 patients (14/22, 64%) were graded mild, 6 patients (6/22, 27%) were moderate and 2 patients (2/22, 9%) were severe according to the National Institutes of Health criteria (Supplementary Table 3).^10^ In the patient subgroup with CR to anti-CD25 mAb therapy, 13 patients (13/37, 35%) developed cGVHD, in which 10 patients (10/13, 77%) were graded mild and 3 patients (3/13, 23%) were moderate. In the PR subgroup, 8 patients (8/16, 50%) developed cGVHD, in which 4 patients (4/8, 50%) were graded mild, 3 patients (3/8, 38%) were moderate and 1 patient (1/8, 12%) was severe. In the NR subgroup, only 1 patient (1/11, 9%) developed cGVHD and was graded severe. There is no significant difference regarding the cGVHD severity distribution pattern between three subgroups (*P*=0.08). For non-steroid-refractory aGVHD patients, PR subgroup patients had the highest cGVHD incidence with significant difference.^8^ Although in our study population, PR subgroup patients held the highest cGVHD incidence but there was no significant difference between these three subgroups (*P*=0.09), which indicates that the effect of response status on cGVHD development in steroid-refractory aGVHD patients after second-line treatment needs more patients included to draw a conclusion.

In this study, overall survival (OS) was defined as time to death from any cause. Non-relapse mortality (NRM) was defined as death in the absence of disease relapse or progression. OS and NRM were calculated from the initiation of anti-CD25 mAb therapy. OS was estimated using the Kaplan–Meier method, survival outcomes between groups were compared with a log-rank test. Probabilities of NRM were estimated by cumulative incidence.

For the entire study patients, an OS rate of 72.9% and a NRM rate of 25.9% were achieved after anti-CD25 mAb treatment at the end of the study with a median follow-up time of 14.1months (range, 0.4–30.5 months). Among the study patients, OS rates of 84.3%, 82.7%, 87.5% and 18.2% and NRM rates of 14%, 14.9%, 12.5% and 81.8% were obtained in patient subgroups with CR+PR, CR, PR and NR response to anti-CD25 mAb therapy, respectively, which implies that achieving response (CR+PR) to anti-CD25 mAb could bring survival benefit and improve the transplant outcome for steroid-refractory aGVHD patients. The OS and NRM curves are shown in Figures 1a and b, and the follow-up data are shown in Supplementary Table 4.

One important consideration regarding the application of anti-CD25 mAb was the potential increased risk of original disease relapse due to a theoretical reduction of the graft-versus-leukemia effect caused by anti-CD25 mAb. However, only one randomized study supports this notion, and in this study daclizumab was used together with steroids for the treatment of aGVHD as a first-line choice.^11^ Moreover, in our patient group only one patient with high-risk factors relapsed and no increased propensity of relapse was observed when anti-CD25 mAb therapy was adopted as a second-line treatment choice. These results are in accordance with a report that anti-CD25 mAb could selectively inhibit activated T cells, without affecting antileukemia capability and therefore did not increase the risk of leukemia relapse.^12^

Although one study showed that anti-CD25 mAb therapy did not impair antiviral activity of recipients,^12^ we did observe 24 (38%, 24/64) of our patients developed opportunistic infections after anti-CD25 mAb administration and in the following 6 months, which comprised of viral (cytomegalovirus and Epstein–Barr virus reactivations) (*n*=11), bacterial (*n*=18) and fungal infections (*n*=2). Furthermore, seven patients died of life-threatening infections during this period. However, the infection rate in our group was still significantly <95% of another report.^13^ In addition, among our patient group, there were still three patients who died of infections beyond 6 months after initiating anti-CD25 mAb therapy. We believed that anti-CD25 mAb contributed little to the infection development in these three patients when considering the terminal elimination half-life of anti-CD25 mAb is nearly 20 days. In general, infection is still the major cause of mortality in patients with aGVHD when treated with anti-CD25 mAb and rational prophylaxis of infection is crucial.

Our report showed that CD25^+^ lymphocyte proportion significantly decreased after anti-CD25 mAb treatment compared with pretreatment, suggesting that CD25^+^ lymphocytes were strongly inhibited (Supplementary Figure 1). One study has shown that donor CD25 expression on CD4^+^ and CD8^+^ T cells increased the risk of GVHD occurrence.^14^ Our results indicated that along with a decrease in CD25^+^ lymphocytes, GVHD would become better controlled.

In summary, humanized anti-CD25 mAb is an effective drug for the treatment of steroid-refractory aGVHD. Furthermore, our study showed that gastrointestinal tract refractory aGVHD showed a significantly better response to anti-CD25 mAb therapy than the skin and liver types. In addition, infection remains the leading cause of death in refractory aGVHD.

## Figures and Tables

**Figure 1:**
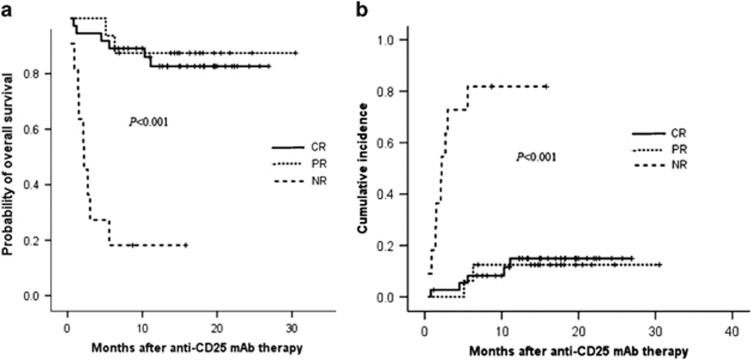
(**a)** Probabilities of OS based on day 28 response in patients with steroid-refractory aGVHD after anti-CD25 mAb therapy. (**b)** Cumulative incidence of NRM based on day 28 response in patients with steroid-refractory aGVHD after anti-CD25 mAb therapy.

**Table 1:** Patient characteristics

*Factor*	n *(%)*
Total no of patients	64
	
*Age*	
Younger than 18 years	8 (12)
Older than 18 years	56 (88)
Median (range), years	35 (13-57)
	
*Gender*	
Male	39 (61)
Female	25 (39)
	
*Diagnosis*	
AML	29 (45)
ALL	17 (27)
CML	6 (9)
MDS	8 (13)
Other	4 (6)
	
*Disease risk*^a^	
Standard	52 (81)
High	12 (19)
	
*Donor type*	
Related	45 (70)
Unrelated	19 (30)
	
*Stem cell source*	
PB	50 (78)
BM	12 (19)
PB+BM	2 (3)
	
*Conditioning*	
Modified BUCY	51 (80)
Modified BUCY+ATG	13 (20)
	
*GVHD prophylaxis*	
CSA+MTX	32 (50)
CSA+MTX+MMF	32 (50)

Abbreviations: ALL, acute lymphocytic leukemia; AML, acute myeloid leukemia; ATG, antithymocyte globulin; BM, bone marrow; BUCY, busulphan+cyclophosphamide; CML, chronic myeloid leukemia; CSA, cyclosporine A; GVHD, graft-versus-host disease; MDS, myelodysplastic syndrome; MTX, methotrexate; MMF, mycophenolate mofetil; PB, peripheral blood.

a Standard risk indicates acute leukemia in CR1 or CR2, CML in first chronic phase, MDS without excess blasts or nonmalignant diseases. High risk indicates others.

## References

[bib1] Holtan SG Pasquini M Weisdorf DJ Acute graft-versus-host disease: a bench-to-bedside update Blood 2014 124 363 373 24914140 10.1182/blood-2014-01-514786 PMC4102709

[bib2] Bordigoni P Dimicoli S Clement L Baumann C Salmon A Witz F Daclizumab, an efficient treatment for steroid-refractory acute-versus-host disease Br J Haematol 2006 135 382 385 16984386 10.1111/j.1365-2141.2006.06321.x

[bib3] Martin PJ Rizzo JD Wingard JR Ballen K Curtin PT Cutler C First- and second-line systemic treatment of acute graft-versus-host disease: recommendations of the American Society of Blood and Marrow Transplantation Biol Blood Marrow Transplant 2012 18 1150 1163 22510384 10.1016/j.bbmt.2012.04.005 PMC3404151

[bib4] Przepiorka D Weisdorf D Martin P Klingemann HG Beatty P Hows J 1994 Consensus conference on acute GVHD grading Bone Marrow Transplant 1995 15 825 828 7581076

[bib5] Bolanos-Meade J Logan BR Alousi AM Antin JH Barowski K Carter SL Phase 3 clinical trial of steroids/mycophenolate mofetil vs steroids/placebo as therapy for acute GVHD: BMT CTN 0802 Blood 2014 124 3221 3227 25170121 10.1182/blood-2014-06-577023 PMC4239331

[bib6] Li J Li X Tan M Lin B Hou S Qian W Two doses of humanized anti-CD25 antibody in renal transplantation MAbs 2009 1 49 55 20046574 10.4161/mabs.1.1.7399 PMC2715186

[bib7] Hockenbery DM Cruickshank S Rodell TC Gooley T Schuening F Rowley S A randomized, placebo-controlled trial of oral beclomethasone dipropionate as a prednisone-sparing therapy for gastrointestinal graft-versus-host disease Blood 2007 109 4557 4563 17244684 10.1182/blood-2006-05-021139

[bib8] MacMillan ML DeFor TE Weisdorf DJ The best endpoint for acute GVHD treatment trials Blood 2010 115 5412 5417 20388871 10.1182/blood-2009-12-258442

[bib9] Wingard JR Majhail NS Brazauskas R Wang Z Sobocinski KA Jacobsohn D Long-term survival and late deaths after allogeneic hematopoietic cell transplantation J Clin Oncol 2011 29 2230 2239 21464398 10.1200/JCO.2010.33.7212 PMC3107742

[bib10] Pavletic SZ Martin P Lee SJ Mitchell S Jacobsohn D Cowen EW Measuring therapeutic response in chronic graft-versus-host disease: National Institutes of Health Consensus Development project on criteria for clinical trials in chronic graft-versus-host disease: IV, Response Criteria Working Group report Biol Blood Marrow Transplant 2006 12 252 266 16503494 10.1016/j.bbmt.2006.01.008

[bib11] Lee SJ Zahrieh D Agura E MacMillan ML Maziarz RT McCarthy PL Jr Effect of up-front daclizumab when combined with steroids for the treatment of acute graft-versus-host disease: results of a randomized trial Blood 2004 104 1559 1564 15138163 10.1182/blood-2004-03-0854

[bib12] Montagna D Yvon E Calcaterra V Comoli P Locatelli F Maccario R Depletion of alloreactive T cells by a specific anti-interleukin-2 receptor P55 chain immuotoxin does not impair *in vitro* antileukemia and antiviral activity Blood 1999 93 3550 3556 10233908

[bib13] Perales MA Ishill N Lomazow WA Weinstock DM Papadopoulos EB Dastigir H Long-term follow up of patients treated with daclizumab for steroid-refractory acute graft-vs-host-disease Bone Marrow Transplant 2007 40 481 486 17618322 10.1038/sj.bmt.1705762

[bib14] Stanzani M Martins SL Saliba RM Bryan S Couriel D Couriel D CD25 expression on donor CD4+or CD8+T cells is associated with an increased risk for graft-versus-host disease after HLA-identical stem cell transplantation in humans Blood 2004 103 1140 1146 12907445 10.1182/blood-2003-06-2085

