# Supplementary material for: Humanized anti-CD25 monoclonal antibody treatment of steroid-refractory acute graft-versus-host disease: a Chinese single-center experience in a group of 64 patients
Source: Blood Cancer J. 2015 Apr 17;5(4):e308–. doi: 10.1038/bcj.2015.33 (PMC4450331; doi:10.1038/bcj.2015.33)
Supplement: Supplementary Figure 1 [file bcj201533x1.doc]

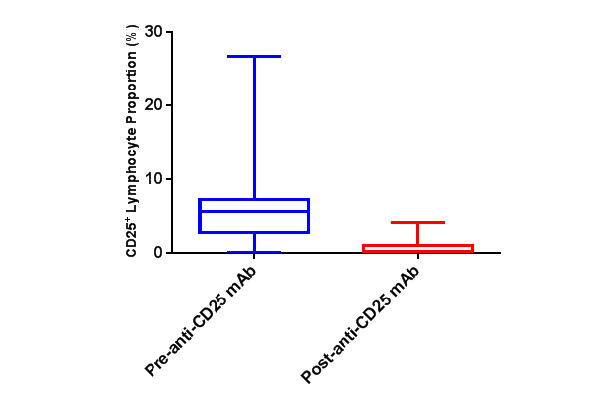


**Supplementary Figure 1.** CD25^+^ lymphocyte proportions before and after anti-CD25 mAb therapy (N=31). Box plots: Boxes show the 25th percentile, median and 75th percentile. Whiskers represent minimum and maximum values.
