# Supplementary material for: Humanized anti-CD25 monoclonal antibody treatment of steroid-refractory acute graft-versus-host disease: a Chinese single-center experience in a group of 64 patients
Source: Blood Cancer J. 2015 Apr 17;5(4):e308–. doi: 10.1038/bcj.2015.33 (PMC4450331; doi:10.1038/bcj.2015.33)
Supplement: Supplementary Table 1 [file bcj201533x2.doc]

**Supplementary Table 1.** Organ-specific staging and overal grading of aGVHD before and after anti-CD25 mAb treatment

| Patient No. | Before anti-CD25 mAb treatment | | | | After anti-CD25 mAb treatment | | | | Therapy response |
| --- | --- | --- | --- | --- | --- | --- | --- | --- | --- |
|  | Skin | GI | Liver | Grade | Skin | GI | Liver | Grade |  |
| 1 | 1 | 4 | 0 | Ⅳ | 0 | 2 | 0 | Ⅱ | PR |
| 2 | 2 | 4 | 1 | Ⅳ | 0 | 2 | 0 | Ⅱ | PR |
| 3 | 0 | 4 | 4 | Ⅳ | 0 | 4 | 4 | Ⅳ | NR |
| 4 | 0 | 3 | 0 | Ⅲ | 0 | 0 | 0 | 0 | CR |
| 5 | 0 | 3 | 2 | Ⅲ | 0 | 3 | 2 | Ⅲ | NR |
| 6 | 2 | 3 | 0 | Ⅲ | 0 | 0 | 0 | 0 | CR |
| 7 | 0 | 4 | 0 | Ⅳ | 0 | 0 | 0 | 0 | CR |
| 8 | 0 | 4 | 2 | Ⅳ | 0 | 4 | 2 | Ⅳ | NR |
| 9 | 2 | 4 | 0 | Ⅳ | 0 | 0 | 0 | 0 | CR |
| 10 | 0 | 4 | 0 | Ⅳ | 0 | 0 | 0 | 0 | CR |
| 11 | 0 | 4 | 0 | Ⅳ | 0 | 0 | 0 | 0 | CR |
| 12 | 0 | 0 | 3 | Ⅲ | 0 | 0 | 2 | Ⅱ | PR |
| 13 | 0 | 4 | 0 | Ⅳ | 0 | 4 | 0 | Ⅳ | NR |
| 14 | 0 | 4 | 0 | Ⅳ | 0 | 0 | 0 | 0 | CR |
| 15 | 0 | 4 | 3 | Ⅳ | 0 | 4 | 3 | Ⅳ | NR |
| 16 | 2 | 3 | 0 | Ⅲ | 0 | 0 | 0 | 0 | CR |
| 17 | 2 | 3 | 0 | Ⅲ | 0 | 0 | 0 | 0 | CR |
| 18 | 1 | 4 | 0 | Ⅳ | 0 | 2 | 0 | Ⅱ | PR |
| 19 | 2 | 2 | 0 | Ⅱ | 0 | 0 | 0 | 0 | CR |
| 20 | 0 | 4 | 0 | Ⅳ | 0 | 0 | 0 | 0 | CR |
| 21 | 0 | 2 | 0 | Ⅱ | 0 | 0 | 0 | 0 | CR |
| 22 | 0 | 0 | 3 | Ⅲ | 0 | 0 | 1 | Ⅰ | PR |
| 23 | 0 | 3 | 0 | Ⅲ | 0 | 0 | 0 | 0 | CR |
| 24 | 3 | 3 | 2 | Ⅲ | 0 | 2 | 2 | Ⅱ | PR |
| 25 | 3 | 4 | 0 | Ⅳ | 0 | 2 | 0 | Ⅱ | PR |
| 26 | 0 | 3 | 2 | Ⅲ | 0 | 0 | 0 | 0 | CR |
| 27 | 4 | 0 | 2 | Ⅳ | 4 | 0 | 2 | Ⅳ | NR |
| 28 | 2 | 0 | 0 | Ⅲ | 0 | 0 | 0 | 0 | CR |
| 29 | 0 | 4 | 0 | Ⅳ | 0 | 0 | 0 | 0 | CR |
| 30 | 0 | 3 | 3 | Ⅲ | 0 | 0 | 0 | 0 | CR |
| 31 | 0 | 4 | 2 | Ⅳ | 0 | 2 | 2 | Ⅱ | PR |
| 32 | 0 | 3 | 0 | Ⅲ | 0 | 0 | 0 | 0 | CR |
| 33 | 0 | 0 | 4 | Ⅳ | 0 | 0 | 4 | Ⅳ | NR |
| 34 | 0 | 4 | 0 | Ⅳ | 0 | 0 | 0 | 0 | CR |
| 35 | 3 | 0 | 0 | Ⅲ | 0 | 0 | 0 | 0 | CR |
| 36 | 0 | 4 | 0 | Ⅳ | 0 | 0 | 0 | 0 | CR |
| 37 | 0 | 3 | 0 | Ⅲ | 0 | 0 | 0 | 0 | CR |
| 38 | 2 | 3 | 2 | Ⅲ | 0 | 2 | 2 | Ⅱ | PR |
| 39 | 0 | 4 | 2 | Ⅳ | 0 | 1 | 1 | Ⅰ | PR |
| 40 | 0 | 3 | 2 | Ⅲ | 0 | 3 | 2 | Ⅲ | NR |
| 41 | 0 | 4 | 0 | Ⅳ | 0 | 1 | 0 | Ⅰ | PR |
| 42 | 0 | 3 | 0 | Ⅲ | 0 | 0 | 0 | 0 | CR |
| 43 | 2 | 4 | 0 | Ⅳ | 0 | 2 | 0 | Ⅱ | PR |
| 44 | 0 | 3 | 0 | Ⅲ | 0 | 0 | 0 | 0 | CR |
| 45 | 0 | 4 | 0 | Ⅳ | 0 | 1 | 0 | Ⅰ | PR |
| 46 | 2 | 3 | 0 | Ⅲ | 0 | 0 | 0 | 0 | CR |
| 47 | 0 | 4 | 0 | Ⅳ | 0 | 2 | 0 | Ⅱ | PR |
| 48 | 0 | 3 | 0 | Ⅲ | 0 | 0 | 0 | 0 | CR |
| 49 | 0 | 3 | 2 | Ⅲ | 0 | 0 | 0 | 0 | CR |
| 50 | 0 | 3 | 0 | Ⅲ | 0 | 0 | 0 | 0 | CR |
| 51 | 0 | 3 | 0 | Ⅲ | 0 | 0 | 0 | 0 | CR |
| 52 | 0 | 4 | 0 | Ⅳ | 0 | 4 | 0 | Ⅳ | NR |
| 53 | 0 | 2 | 0 | Ⅱ | 0 | 0 | 0 | 0 | CR |
| 54 | 2 | 3 | 0 | Ⅲ | 0 | 0 | 0 | 0 | CR |
| 55 | 0 | 4 | 3 | Ⅳ | 0 | 4 | 3 | Ⅳ | NR |
| 56 | 2 | 3 | 0 | Ⅲ | 0 | 0 | 0 | 0 | CR |
| 57 | 2 | 3 | 0 | Ⅲ | 0 | 0 | 0 | 0 | CR |
| 58 | 0 | 3 | 0 | Ⅲ | 0 | 0 | 0 | 0 | CR |
| 59 | 0 | 4 | 0 | Ⅳ | 0 | 4 | 0 | Ⅳ | NR |
| 60 | 0 | 4 | 0 | Ⅳ | 0 | 1 | 0 | Ⅰ | PR |
| 61 | 2 | 4 | 0 | Ⅳ | 0 | 0 | 0 | 0 | CR |
| 62 | 0 | 4 | 0 | Ⅳ | 0 | 0 | 0 | 0 | CR |
| 63 | 0 | 4 | 0 | Ⅳ | 0 | 0 | 0 | 0 | CR |
| 64 | 0 | 0 | 3 | Ⅳ | 0 | 0 | 2 | Ⅱ | PR |

Abbreviations: CR = complete response; PR = partial response; NR = no response;

GI=gastrointestinal tract.
