# Supplementary material for: Humanized anti-CD25 monoclonal antibody treatment of steroid-refractory acute graft-versus-host disease: a Chinese single-center experience in a group of 64 patients
Source: Blood Cancer J. 2015 Apr 17;5(4):e308–. doi: 10.1038/bcj.2015.33 (PMC4450331; doi:10.1038/bcj.2015.33)
Supplement: Supplementary Table 2 [file bcj201533x3.doc]

**Supplementary Table 2.** Response result of steroid-refractory aGVHD patients to anti-CD25 mAb treatment based on numbers of involved organs

| Response  group | Single-organ Involved | | | Two-organs Involved | | | Three-organs Involved |
| --- | --- | --- | --- | --- | --- | --- | --- |
|  | Skin | GI | Liver | Skin+GI | Skin+Liver | GI+Liver | Skin+GI+Liver |
| CR | 2 | 22 | 0 | 10 | 0 | 3 | 0 |
| PR | 0 | 4 | 3 | 4 | 0 | 2 | 3 |
| NR | 0 | 3 | 1 | 0 | 1 | 6 | 0 |

Abbreviations: GI=gastrointestinal tract.
