# Supplementary material for: Humanized anti-CD25 monoclonal antibody treatment of steroid-refractory acute graft-versus-host disease: a Chinese single-center experience in a group of 64 patients
Source: Blood Cancer J. 2015 Apr 17;5(4):e308–. doi: 10.1038/bcj.2015.33 (PMC4450331; doi:10.1038/bcj.2015.33)
Supplement: Supplementary Table 3 [file bcj201533x4.doc]

**Supplementary Table 3.** cGVHD profile in steroid-refractory aGVHD patients after anti-CD25 mAb treatment

| Response Group | cGVHD Grading | | |
| --- | --- | --- | --- |
|  | Mild | Moderate | Severe |
| CR | 10 | 3 | 0 |
| PR | 4 | 3 | 1 |
| NR | 0 | 0 | 1 |

Abbreviations: Response Group = Steroid-refractory aGVHD patient groups classified by the response to anti-CD25 mAb treatment
