# Supplementary material for: Humanized anti-CD25 monoclonal antibody treatment of steroid-refractory acute graft-versus-host disease: a Chinese single-center experience in a group of 64 patients
Source: Blood Cancer J. 2015 Apr 17;5(4):e308–. doi: 10.1038/bcj.2015.33 (PMC4450331; doi:10.1038/bcj.2015.33)
Supplement: Supplementary Table 4 [file bcj201533x5.doc]

**Supplementary Table 4.** Follow-up data of patients

| Patient No. | Secondary infection during treatment | cGVHD onset time after anti-CD25 therapy (day) | cGVHD grading | cGVHD involved organ | Treamtment of chronic GVHD | Surival time after anti-CD25 therapy (day) | The cause of death |
| --- | --- | --- | --- | --- | --- | --- | --- |
| 1 | Pneumonia;Intestinal infection | 421 | Moderate | Liver,Mouth | Corticosteroid, Tacrolimus | 916+ | No |
| 2 | Pneumonia;Intestinal infection | 280 | Mild | Eye | No | 740+ | No |
| 3 | CMV reactivation;Pneumonia; Intestinal infection | No | No | No | No | 89 | GVHD |
|
| 4 | No | No | No | No | No | 730+ | No |
| 5 | CMV reactivation;Pneumonia | No | No | No | No | 46 | Pneumonia |
| 6 | No | 318 | Mild | Skin | CsA | 669+ | No |
| 7 | CMV reactivation | No | No | No | No | 35 | Relapse |
| 8 | IFI | No | No | No | No | 12 | IFI |
| 9 | No | No | No | No | No | 634+ | No |
| 10 | No | No | No | No | No | 669+ | No |
| 11 | No | No | No | No | No | 661+ | No |
| 12 | IFI | 179 | Mild | Skin,Mouth, Eye | Tacrolimus | 652+ | No |
| 13 | No | No | No | No | No | 67 | GVHD |
| 14 | No | 235 | Mild | Mouth | No | 591+ | No |
| 15 | Pneumonia | No | No | No | No | 41 | GVHD |
| 16 | No | 109 | Mild | Skin | No | 135 | IFI |
| 17 | CMV reactivation;Pneumonia; Intestinal infection | 231 | Moderate | Lung | Corticosteroid, Tacrolimus | 308 | Pneumonia |
| 18 | No | 354 | Mild | Skin | CsA | 616+ | No |
| 19 | No | No | No | No | No | 600+ | No |
| 20 | No | No | No | No | No | 590+ | No |
| 21 | No | No | No | No | No | 585+ | No |
| 22 | No | 120 | Severe | Skin,Liver | Corticosteroid,CsA,MTX | 189 | GVHD |
| 23 | No | No | No | No | No | 547+ | No |
| 24 | No | No | No | No | No | 544+ | No |
| 25 | No | 173 | Moderate | Skin,Mouth, Eye | Corticosteroid, Tacrolimus | 589+ | No |
| 26 | No | No | No | No | No | 333 | Pneumonia |
| 27 | Pneumonia | 121 | Severe， (overlap syndrome) | Mouth,Eye,Skin,Musculoskeletal | CsA,MTX,Corticosteroid, Mycophenolate mofetil | 168 | Pneumonia |
| 28 | No | 234 | Moderate | Skin,Musculoskeletal,Mouth | Corticosteroid, Tacrolimus | 511+ | No |
| 29 | No | 180 | Mild | Skin | No | 448+ | No |
| 30 | No | No | No | No | No | 479+ | No |
| 31 | CMV reactivation;Bacterial septicemia | No | No | No | No | 512+ | No |
| 32 | No | No | No | No | No | 549+ | No |
| 33 | Pneumonia | No | No | No | No | 63 | Pneumonia |
| 34 | CMV reactivation | 286 | Mild | Skin,Mouth, Eye | Corticosteroid, Tacrolimus | 808+ | No |
| 35 | No | 209 | Moderate | Skin,Mouth | Tacrolimus | 682+ | No |
| 36 | No | No | No | No | No | 403+ | No |
| 37 | No | 168 | Mild | Mouth, Eye | Corticosteroid, CsA | 772+ | No |
| 38 | Intestinal infection | No | No | No | No | 430+ | No |
| 39 | No | No | No | No | No | 490+ | No |
| 40 | Pneumonia | No | No | No | No | 475+ | No |
| 41 | Pneumonia | No | No | No | No | 154 | Pneumonia |
| 42 | No | No | No | No | No | 531+ | No |
| 43 | No | No | No | No | No | 531+ | No |
| 44 | EBV reactivation; CMV reactivation | No | No | No | No | 398+ | No |
| 45 | No | No | No | No | No | 450+ | No |
| 46 | No | No | No | No | No | 452+ | No |
| 47 | No | No | No | No | No | 415+ | No |
| 48 | No | No | No | No | No | 403+ | No |
| 49 | No | No | No | No | No | 169 | Pneumonia |
| 50 | No | No | No | No | No | 370+ | No |
| 51 | EBV reactivation; CMV reactivation | 157 | Mild | Eye | Tacrolimus | 382+ | No |
| 52 | Intestinal infection;EBV reactivation; CMV reactivation | No | No | No | No | 82 | TMA |
| 53 | No | No | No | No | No | 331+ | No |
| 54 | No | 181 | Mild | Liver | Corticosteroid, Tacrolimus | 299+ | No |
| 55 | Intestinal infection; EBV reactivation; CMV reactivation | No | No | No | No | 26 | GVHD |
| 56 | No | No | No | No | No | 277+ | No |
| 57 | No | No | No | No | No | 243+ | No |
| 58 | No | No | No | No | No | 224+ | No |
| 59 | Pneumonia | No | No | No | No | 262+ | No |
| 60 | Pneumonia | 215 | Moderate, (overlap syndrome) | Gastrointestinal | Tacrolimus，MTX，CTX | 441+ | No |
| 61 | No | 212 | Mild | Skin,Eye | Corticosteroid, Tacrolimus | 370+ | No |
| 62 | Bacterial septicemia | No | No | No | No | 24 | Bacterial septicemia |
| 63 | CMV reactivation;Pneumonia; Intestinal infection | 150 | Mild | Gastrointestinal | Tacrolimus | 203+ | No |
| 64 | No | 180 | Mild | Skin | No | 206+ | No |

Abbreviations: IFI = invasive fungal infections; CNSL =central nervous system leukemia; TMA=thrombotic microangiopathy; cGVHD=chronic graft-versus-host disease.
